# Breast Health Experiences in Women with Cerebral Palsy: A Qualitative Approach

**DOI:** 10.1089/whr.2020.0115

**Published:** 2021-06-28

**Authors:** Linda Ehrlich-Jones, Jordyn Durkin, Rachel Byrne, Allison Todd, Judith Panko Reis, Judith Wolfman, Deborah Gaebler-Spira, Christina Marciniak

**Affiliations:** ^1^ Center for Rehabilitation Outcomes Research, Shirley Ryan AbilityLab, Chicago, Illinois, USA.; ^2^ Department of Physical Medicine and Rehabilitation, Northwestern University Feinberg School of Medicine, Chicago, Illinois, USA.; ^3^ Division of Pulmonary and Critical Care Medicine, Northwestern University Feinberg School of Medicine, Chicago, Illinois, USA.; ^4^ Cerebral Palsy Foundation, New York, New York, USA.; ^5^ Division of Pediatric Orthopedics, Columbia University Medical Center, New York-Presbyterian Morgan Stanley Children's Hospital, New York, New York, USA.; ^6^ Access Living, Chicago, Illinois, USA.; ^7^ Department of Radiology, Northwestern University Feinberg School of Medicine, Chicago, Illinois, USA.; ^8^ Lynn Sage Breast Center, Chicago, Illinois, USA.; ^9^ Department of Pediatrics, Northwestern University Feinberg School of Medicine, Chicago, Illinois, USA.; ^10^ Department of Pediatrics, and Shirley Ryan AbilityLab, Chicago, Illinois, USA.; ^11^ Department of Neurology, Northwestern University Feinberg School of Medicine, Chicago, Illinois, USA.; ^12^ Attending Physician, Shirley Ryan AbilityLab, Chicago, Illinois, USA.

**Keywords:** breast health, preventive medicine, disability

## Abstract

***Background:*** All women, regardless of disability status, should receive screening for breast cancer. In 2010, only 61.4% of women with disabilities (WWD) received a mammogram in the past 2 years compared to 75% of women without disabilities. The purpose of this study is to explore breast cancer screening experiences of women with cerebral palsy (CP) with the aim of identifying factors that could improve screening rates for WWD.

***Methods:*** Thirty women with CP, 22–72 years of age, residing in New York, Chicago, or Los Angeles areas participated in individual or group interviews about breast health. Twenty-five of the participants identified themselves as white, and one self-identified as Hispanic or Latina. Facilitators used a semistructured guide across the three sites. Qualitative analysis utilized an iterative coding process to generate themes related to breast health.

***Results:*** We identified six predominant themes in these interviews, which revolved around physical, environmental, and emotional barriers and facilitators. Within each theme, we identified subthemes. Physical barriers included the most highly identified subthemes of age, pain, holding breath, holding still, spasticity, standing, fatigue, and positioning. Self-advocacy and communication between the health care professional and the patient were the most common subthemes identified among the emotional facilitators.

***Conclusion:*** Women with CP perceive a variety of issues impacting breast health. These findings are multifaceted and suggest that improving screening rates for women with CP should address these barriers and facilitators.

## Introduction

Screening for breast cancer is imperative for all women, including women with disabilities (WWD). Although frequency and age at which to begin screening differ, all US health care organizations include mammography in their first-line screening recommendations.^1–3^ However, according to the National Health Interview Survey completed in 2010, only 61.4% of WWD received a mammogram in the previous 2 years^4^ compared to 75% of women without disabilities.

Nandam et al. found that 65% of women with cerebral palsy (CP), older than 40 years, had a mammogram within the past 2 years and 47.5% had screening mammography.^5^ This lower rate of screening in WWD has been attributed to a number of factors, including a lack of knowledge among health care providers working in mammography, as well as structural barriers at the facilities providing these services.^6^

Breast cancer may be detected earlier using mammography, often when treatment is more effective. Routine mammography screening can reduce breast cancer deaths by up to 20%.^7^ A recent study reported that beginning annual screening at age 40 decreases mortality at 10-year follow-up, and in women 50–69 years of age undergoing mammography, breast cancer mortality rates may be reduced as much as 40%.^8^ It is imperative that WWD are encouraged to undergo breast health screening. This appears to be particularly true for women with CP who have been reported to have a three times higher mortality rate from breast cancer than the general US population.^9^

Although not specifically studied in women with CP, WWD in one study reported that their decisions regarding surgery and chemotherapy with breast cancer were impacted by how these treatments might impact the use of their arms for mobility.^10^ These findings support the need for early and timely follow-up screening for breast cancer in women with CP.

CP is a developmental neurological disorder characterized by abnormal motor function, causing difficulty with movement and posture, resulting in varying abilities and physical disabilities.^11^ CP is reported to affect 764,000 children and adults in the United States.^11^

The prevalence of CP for females is 2.6/1,000 births versus 3.1/1,000 births for males.^12^ Standing balance and the ability to transfer can be impaired in individuals with CP even if that person ambulates in the community or at home. Standard positioning for mammography includes standing upright, remaining still, and holding one's breath. Therefore, women with CP find mammography challenging due to their diminished control and coordination of muscles, fluctuating muscle tone, balance, posture, and strength.^13^

Prior studies have investigated barriers women face with breast cancer screening, but few studies have explored the experiences of women with CP during such procedures.^5^ The purpose of this study was to explore the breast cancer screening experiences of women with CP with the aim of increasing health care professionals' knowledge regarding the care needs of WWD, and ultimately improving screening rates in this population.

## Materials and Methods

Women who participated in the Transforming the Healthcare of Women with Disabilities project were included in this phenomenological study. Participants volunteered to participate in qualitative interviews at the academic medical centers participating in this study across the United States, after each center received study approval by their respective Institutional Review Boards.

Women, 18–89 years of age, who were able to speak and understand English and who had been diagnosed with CP were eligible to participate. Recruitment methods varied between sites. Some participants had previously participated in an online survey administered by this research group. One site had a pre-existing electronic research registry. The other sites used provider referral, clinic flyers, and flyers at a local nonprofit serving people with CP to recruit participants.

Study team members obtained informed consent from all eligible participants before interview participation and confirmed a convenient time for the participant and interviewer to meet individually, through focus groups or providing individual phone interviews. The reason for having three different methods was to allow options for participants who found it hard to travel to an in-person focus group.

Stipends were provided to accommodate participants for the time and travel associated with completing the qualitative interview. Participants were recruited over a 5-month period from August 2015 to December 2015.

Individual or group interviews lasted no longer than 90 minutes; 15–60 minutes were spent discussing breast health depending on the demographics of the group and their overall experiences. Each of the six focus groups included up to three women participating at one of the centers or individual interviews were conducted in person or over the telephone, based on the participant's needs.

A total of 10 semistructured qualitative questions were used across four key content domains to facilitate the focus groups: (1) planning, (2) accessibility of services, (3) appointment experience, and (4) recommendations for improving care. The interviews consisted of questions regarding women's health and participants' experiences with health care providers. Specifically, interview questions focused on gynecologic, reproductive, sexual and obstetric care, and breast health care issues. The moderator had a set of probing questions to help facilitate the conversation and participant recall, which were asked in both the focus group sessions and individual interviews. The subject of this report is limited to content referencing breast health.

Demographic and mobility information were collected from participants. Mobility was rated using the Gross Motor Functional Classification System (GMFCS), which ranges from I—walks without limitations to V-transported in a manual wheelchair with limited axial antigravity and limb control.^14^

The focus groups were facilitated by a therapist with 12 years of clinical and research experience, a social worker with 10 years of research experience, and a therapist with 25 years of clinical and research experience. The moderator used a semistructured format to guide the interviews. Discussion templates were developed by expert knowledge as well as prior research in this area. At least two researchers attended each session and took notes on nonverbal behavior, group dynamics, and emergent themes.

Audio recordings of the sessions were captured for transcription verbatim for analysis. During the interviews, only the first names were used and subsequently de-identified before data analysis.

Transcripts were coded and analyzed using a thematic approach to summarize responses. The coding was undertaken by an iterative process starting with the semistructured interview questions. Two researchers (L.E.J. and J.D.) coded the transcripts separately and then met to compare and resolve any discrepancy through discussion. Data were categorized into relevant themes of barriers and facilitators to breast health screening.

## Results

Thirty women with CP, 22–72 years of age, residing in New York, Chicago, or Los Angeles areas provided information on breast health during individual or group interviews, which covered a range of women's health issues (Table 1). Thirteen individual interviews and a total of six focus groups were conducted across all sites between October 2015 and February 2016; one subject participated in a group interview on Skype. Fifty percent of the women were 40 years of age or older.

**Table 1.** Demographics and Interview Characteristics

No. of subjects	*n* = 30	
Age (years): mean, (SD), range	39.23 (years) 11.99 (SD)	22–72 (years)
Gross motor function classification system	*n* = 30	
Level I	2	6.67%
Level II	12	40%
Level III	7	23.33%
Level IV	9	30%
Level V	0	0%
Race/Ethnicity	*n* = 30	
African American/Black	3	10%
White	25	83.33%
Asian	1	3.33%
Hispanic	1	3.33%
Interview method	*n* = 30	
Phone	9	30%
In person	21	70%
Site	*n* = 30	
New York	15	50%
Chicago	9	30%
Los Angeles	6	20%

SD, standard deviation.

The GMFCS for this sample ranged from one to four. Twelve of the 30 participants were rated a GMFC II, meaning they walk in most settings, but need a hand rail to be able to climb stairs, and 9 of the 30 women were rated as four, meaning their walking ability was severely limited (in need of physical assistance) and in need of a power wheelchair most of the time.^14^

Barriers and facilitators for mammography for WWD each fell into three main categories: physical, environmental, and emotional (Tables 2 and 3).

**Table 2.** Barriers to Mammography

Theme	Subtheme with selected quote^a^	Count
Physical	Age	2
	“I have not gotten a mammogram, I got an ultrasound. Yeah, it's not until you're 35 or 40, so I've done ultrasound.”	
	Discomfort/pain	2
	“I mean she had my breast pushed up and it hurt, it hurt a lot, it was very uncomfortable.”	
	Holding breath	1
	“And the other thing they do is like oh okay, hold it, and then they mosey back to whatever, and they say don't breathe, and then they mosey back, and it's like okay, I haven't been breathing now for 25 seconds, take the damn image.”	
	Holding still	5
	“I had trouble holding still, I've only had it done once because it was so uncomfortable.”	
	Spasticity	3
	“I'm in a standing position, this is my weak arm. It's spastic, if somebody touches it, it will go into the movement.”	
	Standing	3
	“What are you doing to help my stamina when I have to stand here for 15 minutes when I can only really stand for 3 minutes at a time?”	
	Fatigue	2
	“Yes, it would be a bit tiring, you know, sometimes you know like…or I would say, you know I would sit down in-between.”	
	Positioning	7
	“They want me to do this weird leaning back thing, and they're just like well hold it.”	
Environmental	Imaging quality	5
	“Because they manipulated my arm, I had to go back in and retake it, because it did not, they couldn't get a good reading.”	
	Insurance coverage	1
	“But then you run into the problem of they don't cover the ultrasound until you've had the mammogram, so there's always that hurdle as well.”	
	Transportation	1
	“I was like okay, we're wasting more time, and I have a ride coming, and they only wait five minutes, and if I miss that ride I'm stuck down here for another two hours, after I've done with the exam. So once again, transportation and timing is always a factor for us.”	
	Health care professional characteristics	6
	“Sometimes they don't wanna listen to you, they have to find out for themselves. Which is good that they find out, but for me that seems like a waste of time if we already know what we need.”	
	Accessibility	4
	“The machine was not steady for me, it didn't go up and down, so it was a horrible experience”	
Emotional	Perception of care received	3
	“It would have been better to have some help, it was horrible. It was a horrible experience.”	
	Perceptions of breast cancer	5
	“Yes, I will definitely take care of it. I don't want breast cancer. I'm not gonna be so ignorant that I absolutely will bury my head in the sand and ignore it, because it's not gonna go away.”	
	Perception of health care providers	2
	“Yes, he was pretty nice. But people, the one thing doctors have is that whole thing about holding still, and people with CP can't really hold still.”	
	Fear	2
	“And I was really afraid that they had found something”	
	Trust	1
	“The medical profession just needs to trust us who are over 40 to know by now what we need.”	
	Health care professional attitude	4
	“The techs, it's like, they're going through the motions, they're just going through the motions.”	
	Health care professional communication	6
	“You have an emotional experience when they're not listening to what you're asking for.”	

^a^ Highlighted cells are subthemes that were most quoted during focus groups.

CP, cerebral palsy.

**Table 3.** Facilitators to Mammography

Theme	Subtheme with selected quote^a^	Count
Physical	Alternative positioning	3
	“They were able to position me in a way that was satisfactory.”	
Environmental	Accessible changing rooms	1
	“They have the changing room.”	
	Alternative imaging	5
	“I would say for someone that's more severe, that can't stand at all, then just do the ultrasound.”	
	Adjustable machine	5
	“‘It went up and down and it tilted a little bit.’ And that surprised me too, cause I didn't know how flexible the machine was, and they were able to tilt it the way they needed to as far as me turning sideways.”	
	Accessibility	1
	“Since living downtown, everything is better. Because this area is more affluent, it's made more so for people with disabilities.”	
Emotional	Self-advocacy	13
	“Okay, so for me, you know over time, things have got easier just because now I've learned to anticipate and tell people what I need, whereas before we would find it out the hard way.”	
	Communication between health care professional and patient	5
	“Especially down here, they're very excited to learn, and they're always so excited to ask me questions of things, because I can articulate what's going on, and they're always in awe.”	

^a^ Highlighted cells are subthemes that were most quoted during focus groups.

The physical barrier most often identified by women was positioning during the screening procedure followed by holding still. The environmental barrier most often identified was health care professional characteristics, such as the health care provider not listening to the needs of the person with a disability, but implementing ineffective procedures (Table 2), while the emotional barriers most often identified were health care professional communication followed by perceptions of breast cancer.

The most reported emotional facilitator was self-advocacy followed by collaborative communication between the health care professional and patient. The environmental facilitators of alternative imaging and adjustable equipment were also identified as helpful. Health care communication was found to be both a barrier and a facilitator.

## Discussion

Although WWD should undergo breast health screening, their prior experiences with mammography may influence whether they seek subsequent screening. We noted that the barriers to screening identified during this study's interview process far outweighed the facilitators. Women in this study indicated a lack of knowledge by health care providers working in mammography regarding positioning needs related to their disability and other procedures required for comfortable and quality imaging. Lopez et al.^6^ identified structural issues at their facilities as a barrier to care.

While many women identified negative components to their experiences with screening, there were several accounts of positive experiences, in particular, related to alternative positioning, accessible changing rooms, self-advocacy, and communication.

As WWD do not receive breast cancer screenings at a rate comparable to women without disabilities, there should be a concerted effort to improve breast cancer screening rates through addressing communication, physical, and emotional barriers to care.^5,13^ Such efforts may be maximized by focusing on the facilitators to mammography identified in this study and others.^5,15^ Training to enhance mammography technologists' knowledge regarding positioning needs for WWD is available at the CP Foundation website.^16^ Ultimately, we hope that by identifying the unique environmental, physical, and emotional needs of WWD, there will be an improvement in knowledge among health care providers and ultimately lead to improved breast cancer screening rates in these women.

Limitations of this study included that our sample may not be representative of women with CP in general. Many of the participants receive their health care at major academic medical centers where disability awareness could be greater or might be better equipped to address the needs of WWD. Therefore, these results may not be generalizable to the general disability population.

In addition, although a focus group guide was used across sites, the different group facilitators may have concentrated on different areas of women's health care during the various interviews. No woman with very severe motor functional impairments (GMFCS V) participated in this study and this group of individuals is most likely to be disadvantaged by access to breast health screening. Inclusion of these women is important in future studies. Also, more severe motor impairments are more likely to be associated with intellectual disability.^17^ Caregivers' perspectives may also be of value in those with significant intellectual disability.

Strengths of this study include that the women were encouraged to speak freely about their experiences with breast cancer screening. There were a variety of age groups included in this sample and although some women were not of age for routine mammograms, they were able to give a personal perspective. Women also had a range of mobility limitations.

## Conclusions

Women with CP experience a variety of barriers and facilitators related to breast health screening. These findings are multifaceted and suggest that screening rates for these women may improve by addressing barriers and promoting facilitators identified by women with CP.

## Abbreviations Used

WWD: women with disabilities
CP: cerebral palsy
GMFCS: Gross Motor Functional Classification System
SD: standard deviation
